# Visual outcomes with two posterior chamber phakic intraocular lenses in myopic patients: a contralateral eye study

**DOI:** 10.3389/fmed.2025.1438569

**Published:** 2025-07-09

**Authors:** Boliang Li, Xun Chen, Mingrui Cheng, I-Chun Lin, Yinjie Jiang, Yadi Lei, Guanghan Xu, Mingwei Li, Zhiwei Mao, Xingtao Zhou, Xiaoying Wang

**Affiliations:** ^1^Eye Ear Nose and Throat Hospital, Fudan University, Shanghai, China; ^2^National Health Commission Key Lab of Myopia (Fudan University), Shanghai, China; ^3^Shanghai Research Center of Ophthalmology and Optometry, Shanghai, China; ^4^State Key Laboratory of Medical Neurobiology and MOE Frontiers Center for Brain Science, Fudan University, Shanghai, China

**Keywords:** PIOL, implantable collamer lens, refractive outcomes, visual quality, myopia

## Abstract

**Purpose:**

Assess the efficacy, safety, and visual quality of two posterior chamber phakic intraocular lenses (PIOL) for myopia correction.

**Methods:**

In this randomized double-blind prospective controlled trial, 38 eyes of 19 myopic patients received phakic refractive (PR) IOL in one eye and implantable collamer lens (EVO ICL) in the opposite eye and were followed up for 1 year. Uncorrected distance visual acuity, corrected distance visual acuity, vault, disk halo size, contrast sensitivity, higher-order aberrations, retinal imaging quality, intraocular scattering, and subjective glare/halo perception were measured.

**Results:**

At 1 year, mean efficacy indices were 1.03 ± 0.16 (PR) and 1.01 ± 0.15 (ICL), and mean safety indices were 1.18 ± 0.07 (PR) and 1.15 ± 0.09 (ICL). The PR group had lower vaults than those of the ICL group at 1 year (342.63 ± 153.94 vs. 470.00 ± 230.31 μm, *P* = 0.010). One month postoperatively, the severity of glare/halo was significantly milder in the PR group than that in the ICL group (*P* = 0.035). However, 1 year postoperatively, no significant differences were observed in the severity of glare/halo between the two groups.

**Conclusion:**

Phakic refractive implantation is a safe and effective treatment option for myopia. The PR group exhibited lower vaults than those of the ICL group. The PR group had milder glare/halo in the early postoperative period compared to the ICL group, though long-term differences were not significant.

## 1 Introduction

Posterior chamber phakic intraocular lenses (PIOLs) have become increasingly popular for myopia correction. The Visian Implantable Collamer Lens (ICL; Staar Surgical Co., Monrovia, CA, United States) is a commonly used PIOL for myopia correction, known for its demonstrated safety and effectiveness (1–3). The Phakic Refractive (PR) IOL (Eyebright Medical Technology, Beijing, China) is a fresh variant of the central hole plate-haptic single-piece PIOL made of acrylate material that incorporates ultraviolet absorbers. Having received registration approval from the National Medical Products Administration of China (Registration Certificate No: 20253160001), it has entered clinical practice as a new therapeutic option for patients with high myopia. The PR has more size options, a larger optical area, and a smaller diopter interval than the EVO ICL. This study aimed to compare the efficacy, safety, and subjective and objective visual quality of the PR and EVO ICL.

## 2 Materials and methods

### 2.1 Participants

This study adhered to the Declaration of Helsinki and was approved by the Ethical Committee Review Board of Fudan University Eye and ENT Hospital (Permit Number: 2019014). Written informed consent was obtained from all participants after a comprehensive explanation of the potential risks and benefits of the study. This double-blind prospective study involved 38 eyes of 19 patients with myopia. One eye was randomly assigned to receive PR implantation, whereas the contralateral eye received EVO ICL implantation. Patients and outcome assessors were masked to lens type allocation to minimize assessment bias, while surgeons remained unavoidably unblinded due to the inherent visual differences between the lenses. All surgeries were performed at the Eye and ENT Hospital of Fudan University (Shanghai, China).

Preoperatively, all patients underwent comprehensive ophthalmic evaluation to meet the surgical criteria. These assessments included uncorrected distance visual acuity (UDVA) and corrected distance visual acuity (CDVA), manifest and cycloplegic refraction, slit-lamp biomicroscopic and fundoscopic examinations, intraocular pressure (IOP, Tonemeterx-10; Canon, Tokyo, Japan), corneal topography (Pentacam, Oculus, Germany), central corneal thickness (CCT, Pentacam), horizontal corneal diameter (white-to-white, WTW, Pentacam), axial length (IOL Master, Carl Zeiss, Germany), anterior chamber depth (ACD, Pentacam), corneal endothelial cell density (ECD, SP-3000P, Topcon Corporation, Japan), wavefront aberrations (i.Profiler Plus, Carl Zeiss, Germany), disk halo size (MonPack One, Metrovision, France), optical coherence tomography (Optovue, United States), and ultrasound biomicroscopy (UBM, Quantel Medical, France).

The study included patients aged 21–45 years, with stable refractive error (change ≤ 0.50 dpt (D)/year) for a minimum of 2 years, myopia ranging from −0.50 to −18.00 D spherical equivalent (SE), astigmatism ≤ 1.50 D, ACD ≥ 2.80 mm, and ECD ≥ 2,000 cells/mm^2^. The exclusion criteria included an anterior chamber angle Grade < III, ocular hypertension, preoperative CDVA < 20/63, history of certain ocular diseases (such as suspected keratectasia, corneal or lens opacity, glaucoma, pseudoexfoliation, pigment dispersion, uveitis, retinal detachment, macular degeneration, or neuroophthalmic disease), prior corneal or intraocular surgeries, history of inflammation or trauma, or systemic disease.

### 2.2 Phakic refractive lens and implantable collamer lens

**FIGURE 1:**
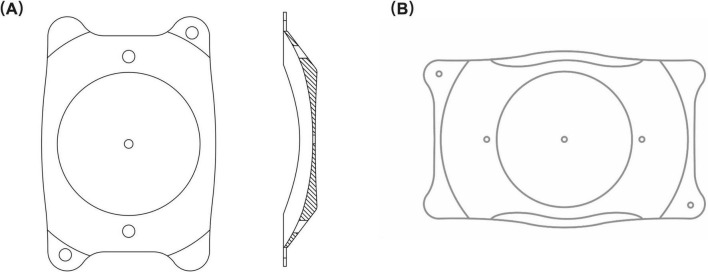
Lens structure. **(A)** The structure of the phakic refractive intraocular lens (PR); **(B)** The structure of the implantable collamer lens (ICL). Both intraocular lenses have a central hole and peripheral positioning holes. The PR is comprised of a central hole size of 370 μm and a peripheral hole size of 500 μm. The central and peripheral hole sizes of the ICL are both 360 μm.

The PR is a plate-haptic single-piece intraocular lens equipped with a 370 μm central hole (Figure 1A). Similar to the EVO ICL, the PR shares the capability of being foldable and can be implanted in the posterior chamber via a 2.8–3.2 mm corneal incision. The lens spans a dioptric power range of −0.50 to −18.00 D with 0.25 D intervals, featuring a high refractive index and large central optic zone with a diameter of 6.0 mm. It offers 10 sizes, varying with 0.3 mm intervals: 11.5, 11.8, 12.1, 12.4, 12.7, 13.0, 13.3, 13.6, 13.9, 14.2 mm. PR power calculation and size selection were performed by the manufacturer (Eyebright Medical Technology).

The lens power step of the EVO ICL (Figure 1B) is 0.25 D from −0.50 to −3.00 D and 0.50 D from −3.00 to −18.00 D. It comes in four sizes: 12.1, 12.6, 13.2, and 13.7 mm. The optic diameter of the EVO ICL varies according to dioptric power, ranging from 4.9 to 5.8 mm. ICL power and size calculations were performed using manufacturer-provided software (Staar Surgical Co).

### 2.3 Surgical technique

An experienced surgeon (XW) performed PR and EVO ICL implantation in all included patients using a previously described one-step technique (4). Before surgery, pupils were dilated with 2.5% phenylephrine and 1% tropicamide. The lens was implanted via a 3 mm temporal corneal incision using an injector cartridge. A moderately viscoelastic surgical agent, 1% sodium hyaluronate, was introduced into the anterior chamber. The lens was positioned in the posterior chamber, and the viscoelastic surgical agent was washed away using a balanced salt solution. Postoperative medications included antibiotics, non-steroidal anti-inflammatory eye drops, steroidal eye drops, and artificial tears.

### 2.4 Follow-up

Patients underwent follow-up at 1 day, 1 week, 1 month, 3 months, and 1 year postoperatively. Assessments included UDVA, CDVA, manifest refractive error, IOP, vault, ECD, axial length, and objective visual quality parameters, such as disk halo size, contrast sensitivity, HOAs, retinal imaging quality, and intraocular scattering. Vault was measured using Pentacam. A written questionnaire was used to evaluate subjective symptoms and interocular differences in each patient at 1 month and 1 year postoperatively. Patients were retrospectively asked about their overall satisfaction with the procedure, willingness to undergo surgery, and whether they would recommend the procedure to others. To minimize bias, the participants were blinded, and examinations were performed and recorded by blinded examiners.

### 2.5 Disk halo size and contrast sensitivity

An experienced technician assessed the disk halo size and contrast sensitivity using a vision monitor. Following established methodologies (5, 6), the disk halo size was evaluated at a 2.5 m distance after 5 min of dark adaptation, using a light source with a luminance of 5 cd/m^2^. A corrective lens was used for preoperative measurements. The static and dynamic contrast sensitivities were evaluated at a distance of 2 m after 5 min of dark adaptation. Following established protocols (6, 7), the visual monitor presented vertical gratings of sine waves at various spatial frequencies (SFs), including 0.5 (SF0.5), 1.1 (SF1.1), 2.2 (SF2.2), 3.4 (SF3.4), 7.1 (SF7.1), and 14.6 (SF14.6) cycles/degree (cpd), and presenting the contrast sensitivity at each SF in dB units.

### 2.6 Assessment of higher-order aberrations

Higher-order aberrations were measured using an i.Profiler Plus (Carl Zeiss, Germany) before and 1 year after surgery. Zernike coefficients were utilized to analyze the spherical aberration (Z_4_^0^), root mean square (RMS) values of total HOAs (third–sixth order), trefoil (Z_3_^–3^, Z_3_^3^), and coma (Z_3_^–1^, Z_3_^1^) for a 5 mm pupil diameter.

### 2.7 Retinal image quality and intraocular scattering measurement

Retinal image quality and intraocular scattering were objectively measured 1 year postoperatively under mesopic conditions using a double-pass optical quality analysis system (OQAS II; Visiometrics, Terrassa, Spain). As previously outlined (8–11), all measurements were performed with a 4 mm aperture. Corrections for a cylindrical diopter of ≥ 0.50 D were made using an external lens, whereas the system automatically corrected spherical diopter. The objective scatter index (OSI) quantifies intraocular scatter by measuring the ratio of light in the peripheral area to the central peak in the acquired double-pass image (12). Lower OSI values indicate better optical quality (11). Five representative indices were derived from the modulation transfer function (MTF) profile for retinal image quality evaluation. The MTF cut-off frequency represents the SF at which the MTF reaches its lowest contrast of 1% (13). Strehl in two-dimensional ratio (SR) denotes the ratio between the aberrated eye and an ideal aberration-free eye in the MTF profiles, ranging from 0 to 1.0 (14). The three OQAS values (OV100%, OV20%, and OV9%) are normalized values of SFs at 0.01, 0.05, and 0.1 MTF values, respectively, reflecting optical quality under commonly used contrast conditions (11).

### 2.8 Statistical analysis

Statistical analyses were performed using SPSS version 26.0 (SPSS Corp., Armonk, NY, United States). Continuous variables are presented as means ± standard deviation, whereas categorical variables are presented as percentages. The Shapiro–Wilk test was used to determine the normal distribution of continuous variables. Based on data characteristics, paired *t*-tests, Wilcoxon signed-rank tests, or generalized estimating equations were used for preoperative, postoperative, and between-group comparisons. The statistical significance of the percentage differences was assessed using the McNemar-Bowker and Wilcoxon signed-rank tests. Differences were considered statistically significant at a *P*-value of < 0.05.

## 3 Results

A total of 38 eyes of 19 patients with myopia (15 women and four men) were included in the study, with one eye randomly receiving PR and the contralateral eye receiving EVO ICL. Preoperative patient statistics and characteristics of the implanted PIOLs are summarized in Table 1. The mean age of the patients was 27.79 ± 5.01 (21–39) years. No significant differences were observed between eyes treated with PR and those treated with ICL in spherical error, cylinder, CDVA, keratometric values, axial length, IOP, ECD, CCT, ACD, WTW, Horizontal STS, Vertical STS, PIOL power, or PIOL size (all *P* > 0.05).

**TABLE 1:** Preoperative patient statistics and characteristics of implanted PIOLs.

Parameters	PR		ICL		*P*
	Mean ± SD	Range	Mean ± SD	Range	
Preoperative spherical error (D)	−7.42 ± 2.09	−11.75, −3.75	−7.33 ± 2.02	−10.25, −4.00	0.794
Preoperative cylinder (D)	−0.63 ± 0.36	−1.50, 0.00	−0.67 ± 0.41	−2.00, −0.25	0.582
Preoperative SE (D)	−7.74 ± 2.12	−12.00, −3.88	−7.66 ± 2.08	−10.63, −4.13	0.810
CDVA (logMAR)	−0.00 ± 0.03	−0.10, 0.10	−0.02 ± 0.04	−0.10, 0.00	0.083
Flat K (D)	42.78 ± 1.40	40.1, 45.3	42.79 ± 1.48	−39.9, 45.6	0.793
Steep K (D)	43.70 ± 1.63	40.2, 46.5	43.77 ± 1.56	40.6, 46.7	0.259
Axial length (mm)	26.79 ± 1.20	24.79, 29.95	26.75 ± 1.30	24.52, 29.02	0.807
IOP (mmHg)	15.03 ± 2.58	11.5, 21.0	14.72 ± 2.55	11.5, 21.8	0.122
ECD (cells/mm^2^)	2748.53 ± 195.09	2502, 3335	2772.74 ± 224.45	2504, 3236	0.715
CCT (μm)	525.42 ± 38.22	470, 588	528.42 ± 37.84	467, 587	0.820
ACD (mm)	3.30 ± 0.31	2.85, 4.15	3.28 ± 0.31	2.80, 4.09	0.285
WTW (mm)	11.62 ± 0.38	11.0, 12.6	11.62 ± 0.36	11.0, 12.5	1.000
Horizontal STS (mm)	11.92 ± 0.39	11.37, 12.79	11.90 ± 0.44	11.10, 12.73	0.572
Vertical STS (mm)	12.49 ± 0.47	11.71, 13.31	12.51 ± 0.48	11.86, 13.32	0.605
PIOL size (mm)	12.94 ± 0.35	12.4, 13.6	12.91 ± 0.40	12.6, 13.7	0.268
PIOL power (D)	−8.63 ± 2.04	−12.00, −5.00	−8.47 ± 2.17	11.50, −4.50	0.628

PIOL, posterior chamber phakic intraocular lens; PR, phakic refractive intraocular lens; ICL, implantable collamer lens; SD, standard deviation; D, diopters; SE, spherical equivalent; CDVA, corrected distance visual acuity; K, keratometric value; IOP, intraocular pressure; ECD, corneal endothelial cell density; CCT, central corneal thickness; ACD, anterior chamber depth; WTW, white-to-white; STS, sulcus-to-sulcus.

### 3.1 Efficacy, safety, and predictability

**FIGURE 2:**
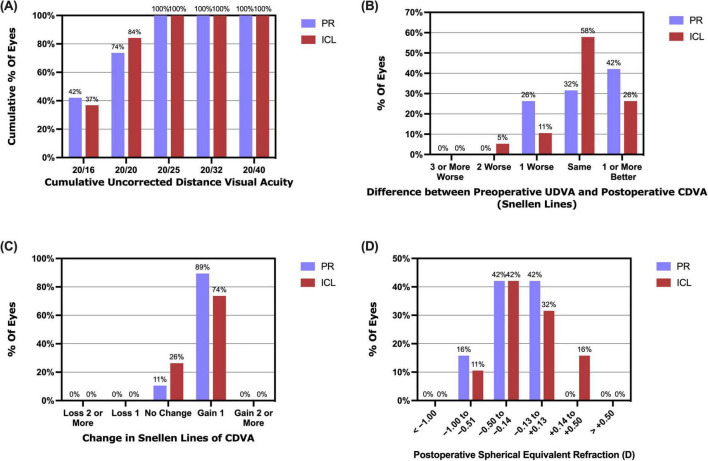
Refractive outcomes at 1 year post-implantation of the phakic refractive intraocular lens (PR) and the implantable collamer lens (ICL). **(A)** The cumulative uncorrected distance visual acuity (UDVA); **(B)** The postoperative UDVA versus preoperative corrected distance visual acuity (CDVA); **(C)** The changes in the lines of CDVA; **(D)** Postoperative spherical equivalent refraction.

At the 1 year follow-up, 14 (73.7%) PR-treated eyes and 16 (84.2%) ICL-treated eyes achieved a UDVA of 20/20 or better, with all eyes in both groups achieving a UDVA of 20/25 or better (Figure 2A). Mean efficacy indices (postoperative UDVA/preoperative CDVA) at 1 day, 1 week, 1 month, 3 months, and 1 year in the PR group were 0.94 ± 0.20, 1.02 ± 0.11, 1.12 ± 0.17, 1.08 ± 0.16 and 1.03 ± 0.16, respectively. The values in the ICL group were 1.00 ± 0.11, 1.07 ± 0.14, 1.10 ± 0.19, 1.10 ± 0.13 and 1.01 ± 0.15, respectively. No significant differences in efficacy indices were observed between the two groups (*P* = 0.573). Additionally, at 1 year postoperatively, 14 (73.7%) eyes in the PR group and 16 (84.2%) eyes in the ICL group had a UDVA equal to or better than the preoperative CDVA (Figure 2B).

All procedures proceeded without incidence, and no intra- or postoperative complications were noted during the 1 year follow-up period. Mean safety indices (postoperative CDVA/preoperative CDVA) at 1 week, 1 month, 3 months, and 1 year in the PR group were 1.16 ± 0.09, 1.24 ± 0.13, 1.20 ± 0.10 and 1.18 ± 0.07, respectively. The ICL group safety indices were 1.16 ± 0.12, 1.21 ± 0.15, 1.16 ± 0.12 and 1.15 ± 0.09, respectively. No significant differences were observed in the safety indices between the two groups (*P* = 0.385). Furthermore, 1 year postoperatively, 17 (89.5%) eyes in the PR group and 14 (73.7%) eyes in the ICL group had gained one line of CDVA, with no eyes in either group experiencing a loss of one or more lines of CDVA (Figure 2C).

**FIGURE 3:**
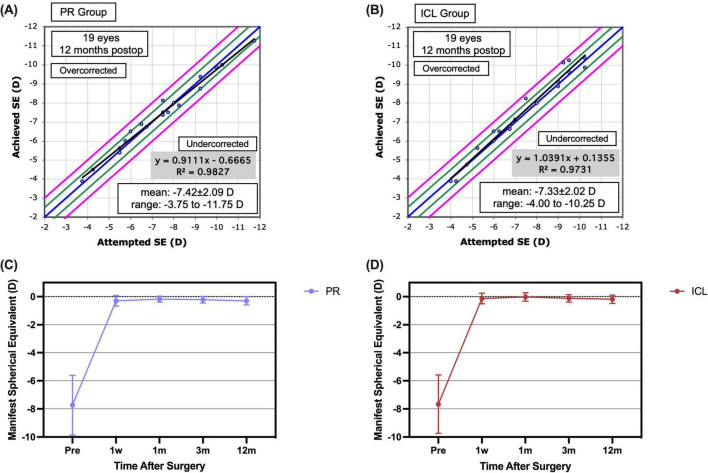
Predictability and stability of the phakic refractive intraocular lens (PR) and the implantable collamer lens (ICL) at 1 year post-implantation. **(A)** The attempted versus achieved spherical equivalent refraction post-implantation of the PR; **(B)** The attempted versus achieved spherical equivalent refraction post-implantation of the ICL; **(C)** The post-implantation manifest spherical equivalent refraction of the PR; **(D)** The post-implantation manifest spherical equivalent refraction of the ICL.

At the 1 year mark postoperatively, the spherical equivalent (SE) for 16 (84.2%) PR-treated eyes and 17 (89.5%) ICL-treated eyes was within ± 0.50 D, and for all eyes, it fell within ± 1.00 D (Figure 2D). The scatter plots in Figures 3A, B illustrate the attempted versus achieved SE correction in the PR and ICL groups (R^2^ = 0.9827 and R^2^ = 0.9731, respectively). The manifest refractive spherical equivalent (MRSE) shifted from −7.74 ± 2.12 D preoperatively to −0.30 ± 0.38 D at 1 week, −0.18 ± 0.21 D at 1 month, −0.22 ± 0.24 D at 3 months, and −0.30 ± 0.29 D at 1 year after PR implantation (Figure 3C). In the ICL group, MRSE changed from −7.66 ± 2.08 D preoperatively to −0.13 ± 0.38 D at 1 week, −0.02 ± 0.30 D at 1 month, −0.12 ± 0.26 D at 3 months, and −0.18 ± 0.30 D at 1 year after ICL implantation (Figure 3D). No significant differences in MRSE were noted between the two groups (*P* = 0.081). No significant changes in MRSE were observed from 1 week to 1 year postoperatively in either the PR or ICL group (*P* = 0.468 and *P* = 0.437, respectively). Moreover, no significant axial elongation was observed at the 1 year mark post-surgery compared with the preoperative state in both the PR and ICL groups (*P* = 0.101 and *P* = 0.834, respectively).

### 3.2 Intraocular pressure, endothelial cell density, and vault

The IOP remained stable for 1 year after surgery, and no significant differences were noted between the two groups (*P* = 0.703). ECD decreased 1.27 ± 5.45% in the PR group and 2.23 ± 8.67% in the ICL group from the preoperative period to 1 year postoperatively. No significant changes were observed in the ECD in the PR (*P* = 0.287) or ICL (*P* = 0.200) groups between the preoperative period and 1 year postoperatively.

After 1 year postoperatively, the mean vault decreased from 354.21 ± 148.19 μm at 1 month to 342.63 ± 153.94 μm in the PR group. Similarly, postoperatively, the mean vault decreased from 500.79 ± 247.68 μm at 1 month to 470.00 ± 230.31 μm in the ICL group. At 1 month and 1 year postoperatively, the vault in the PR group was significantly lower than that in the ICL group (*P* = 0.004 and *P* = 0.010, respectively). No significant differences were observed in the vault measurements between 1 month and 1 year postoperatively in either group (both *P* > 0.05).

### 3.3 Visual disturbances and satisfaction

**FIGURE 4:**
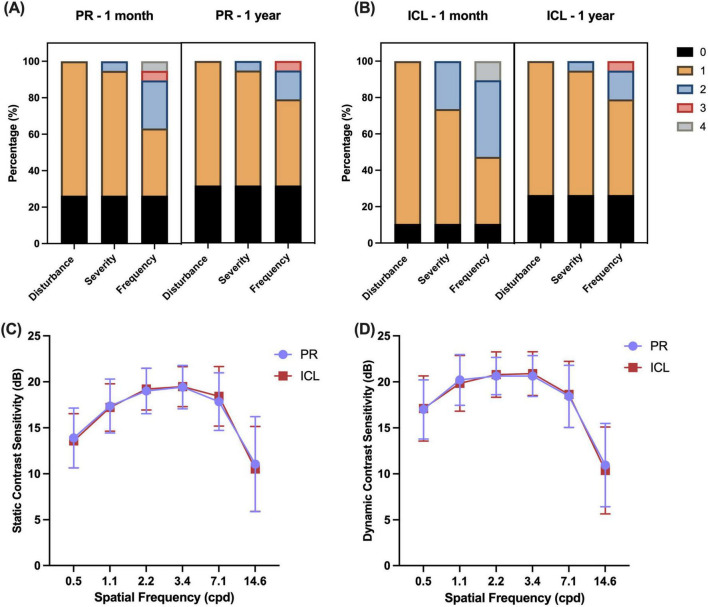
Nocturnal visual disturbances and contrast sensitivity after phakic refractive intraocular lens (PR) and implantable collamer lens (ICL) implantation. **(A)** Glare/halo disturbances at 1 month and 1 year after PR implantation; **(B)** Glare/halo disturbances at 1 month and 1 year after ICL implantation; **(C)** Static contrast sensitivities of the two lenses after implantation; **(D)** Dynamic contrast sensitivities of the two lenses after implantation. Disturbance scores: 0, no; 1, yes. Severity scores: 0, none; 1, mild; 2, moderate; 3, severe. Frequency scores: 0, none; 1, occasionally; 2, sometimes; 3, most of the time; and 4, always.

The nocturnal visual disturbances after PR and ICL implantation are summarized in Figures 4A, B. One month after surgery, the prevalence of glare or halo was not significantly different between the PR and ICL groups (*P* = 0.453). However, severity was significantly milder in the PR group than that in the ICL group (*P* = 0.035). One year postoperatively, no significant differences were observed in the prevalence, severity, or frequency of glare/halo between the PR and ICL groups (all *P* > 0.05). At 1 month postoperatively, 11 (57.9%) patients felt that the ICL had a heavier glare/halo than the PR; however, by 1 year postoperatively, 14 (73.7%) patients felt that there was no difference in the glare/halo between the two eyes. The mean satisfaction score of the 19 enrolled patients was 9.16 ± 0.76 (range, 8–10). All patients expressed satisfaction with the surgery, indicated their willingness to undergo the same surgery again, and would recommend the procedure to others experiencing similar discomfort.

### 3.4 Disk halo size and contrast sensitivity

The disk halo size (Table 2) was significantly decreased 1 year postoperatively compared with the preoperative size in both groups (both *P* < 0.001). However, no significant difference was observed in the decrease in halo radius between the two groups (*P* = 0.166). No significant differences were observed in the static and dynamic contrast sensitivities (Figures 4C, D) between the two groups (all *P* > 0.05).

**TABLE 2:** Comparison of disk halo size and higher-order aberrations between groups.

Parameters		PR group	ICL group	*P* ^a^
**Disk halo size (arcmin)**				
	Pre	105.79 ± 29.87	103.16 ± 25.62	0.485
	1 year	86.84 ± 31.63	87.89 ± 26.16	0.751
	Δ	−18.95 ± 8.09	−15.26 ± 8.41	0.166
	*P* ^b^	< 0.001*	<0.001*	–
**Coma (μm)**				
	Pre	0.12 ± 0.06	0.12 ± 0.05	0.844
	1 year	0.11 ± 0.05	0.13 ± 0.07	0.261
	Δ	−0.01 ± 0.07	0.01 ± 0.07	0.3
	*P* ^b^	0.485	0.682	–
**Trefoil (μm)**				
	Pre	0.13 ± 0.06	0.12 ± 0.07	0.298
	1 year	0.19 ± 0.10	0.18 ± 0.10	0.648
	Δ	0.06 ± 0.12	0.06 ± 0.12	0.958
	*P* ^b^	0.025*	0.038*	–
**Spherical aberration (μm)**				
	Pre	0.04 ± 0.06	0.04 ± 0.07	0.74
	1 year	0.03 ± 0.07	0.03 ± 0.05	0.763
	Δ	−0.01 ± 0.06	−0.01 ± 0.06	0.555
	*P* ^b^	0.858	0.492	–
**Total HOAs (μm)**				
	Pre	0.23 ± 0.08	0.24 ± 0.06	0.622
	1 year	0.27 ± 0.12	0.28 ± 0.10	0.783
	Δ	0.04 ± 0.10	0.04 ± 0.11	0.981
	*P* ^b^	0.1	0.129	–

^a^Signifies comparison between PR group and ICL group.

^b^Represents preoperative and postoperative comparisons. ICL, implantable collamer lens; PR, phakic refractive intraocular lens; RMS, root mean square; HOAs, higher-order aberrations; Δ, the value at 1 year − the value before surgery.

*P < 0.05.

### 3.5 Wavefront aberrations

Following surgery, the trefoil for the 5 mm pupil diameter significantly increased in both groups (both *P* < 0.05). No significant differences were noted between the two groups in coma, spherical aberration, total HOAs, or postoperative changes in all HOAs (Table 2).

### 3.6 Retinal image quality and intraocular scattering

The PR group demonstrated a significantly lower MTF cutoff and SR than those in the ICL group (*P* = 0.041 and *P* = 0.023, respectively, Table 3). Although the PR group displayed slightly lower OV100%, OV20%, and OV9% and a higher OSI than those of the ICL group, these differences were not statistically significant.

**TABLE 3:** Postoperative OQAS parameters between groups (Mean ± SD).

OQAS parameters	PR group	ICL group	*P*
OSI	1.15 ± 0.51	0.95 ± 0.42	0.107
MTF cut-off (cpd)	30.08 ± 6.28	34.99 ± 8.98	0.041*
SR	0.17 ± 0.03	0.20 ± 0.05	0.023*
OV100%	1.00 ± 0.19	1.14 ± 0.29	0.085
OV20%	0.73 ± 0.18	0.83 ± 0.22	0.086
OV9%	0.44 ± 0.11	0.50 ± 0.14	0.117

OQAS, optical quality analysis system; SD, Standard deviation; PR, phakic refractive intraocular lens; ICL, implantable collamer lens; OSI, objective scatter index; MTF, modulation transfer function; CPD, cycle per degree; SR, Strehl in two dimensions ratio; OV, OQAS value;

*P < 0.05.

## 4 Discussion

Phakic refractive is a novel PIOL, and to our knowledge, its clinical outcomes have not been investigated. In various studies, EVO ICL implantation has consistently shown safe, reliable, and predictable outcomes for myopia correction (15, 16), ensuring high and stable postoperative visual quality (17, 18). In this study, we assessed the safety, efficacy, stability, predictability, and subjective/objective visual quality of the PR using the ICL as a comparison.

A literature review by Packer revealed a mean efficacy index of 1.04, with 90.8% achieving SE within ± 0.50 D and 98.7% achieving SE within ± 1.00 D across 1,905 eyes, averaging a follow-up of 12.5 months (19). Another recent review reported that post-ICL implantation, efficacy and safety indices frequently surpass 1.0 (1). Our results confirm that implantation surgery using the PR is effective. However, the predictability of the PR appears slightly inferior to that of the ICL, with PR-treated eyes displaying a tendency toward under correction. This observation may explain the postoperative superior UDVA in the eyes of patients treated using the ICL. PR has smaller diopter intervals and should theoretically correct myopia more precisely. This may be due to the short clinical application time and the lack of experience with power calculations. In addition, a smaller vault may contribute slightly to the more myopic results obtained in the PR group. Therefore, manufacturers may be able to achieve more accurate corrections in the future by further optimizing the power and size calculation formula.

No patient in either group experienced loss of CDVA 1 year postoperatively. The mean safety indices at 1 year were 1.18 ± 0.07 and 1.15 ± 0.09 in the PR and ICL groups, respectively. These results align with those of the review by Packer, reporting a mean safety index of 1.15 across 4,196 eyes with an average follow-up of 14.0 months (19). No statistically significant difference was observed between the preoperative and postoperative ECD (*P* = 0.230). Wei et al. (20), Sachdev et al. (21) reported no significant changes in ECD between the preoperative period and 6 months post-surgery, indicating that the surgical procedure with both lenses minimally affected ECD. These findings suggest that the PR implantation is safe.

In our study, no significant changes were noted in the MRSE from 1 week to 1 year postoperatively in either the PR or ICL groups (*P* = 0.468 and *P* = 0.437, respectively). Additionally, no significant axial elongation was observed at 1 year postoperatively compared with the preoperative measurements in both the PR and ICL groups (*P* = 0.101 and *P* = 0.834, respectively). These findings indicate the stability of both the PR and ICL implantations postoperatively.

Phakic refractive eyes exhibited lower vaults than ICL eyes, with mean vaults measuring 342.63 ± 153.94 μm in the PR group and 470.00 ± 230.31 μm in the ICL group at 1 year post-surgery. The ideal vault range (250–750 μm) was achieved in 12 (63.2%) eyes in the PR group and 14 (73.7%) eyes in the ICL group. However, if we increase the vault by 100 μm, 18/19 (94.7%) of the PR eyes will be within the ideal vault range. Therefore, manufacturers should optimize their size selection strategy and choose a larger lens to achieve higher vaults in future patients. Similar to previous studies, our study revealed a gradual decrease in the vault over the long term (22, 23).

Assessing optical quality after refractive surgery often involves evaluating glare and halo symptoms, which are significant concerns for both patients and refractive surgeons. The questionnaire results revealed that the severity of the glare/halo in the PR group was milder than that in the ICL group 1 month postoperatively, and the difference was significant (*P* = 0.035), potentially due to the larger optical area of the PR than that of the ICL. However, 1 year after surgery, the severity of the glare/halo in the ICL group had decreased significantly (*P* = 0.008), and there was no longer a significant difference between the two groups (*P* = 0.655). This may be because neuroadaptation becomes more prominent with time, leading to a gradual decrease in glare/halo after surgery (5).

One year post-surgery, mean halo radii in the PR and ICL groups were 86.84 ± 31.63 arcmin and 87.89 ± 26.16 arcmin, respectively, aligning with previous findings (6, 7, 24). Both lenses exhibited a significant decrease in halo radii 1 year postoperatively (both *P* < 0.001), with no significant difference between them (*P* = 0.166). Similarly, Chen et al. (5) noted a substantial reduction in disk halo size at 1 and 3 months after EVO ICL implantation. Conversely, Zhao et al. (25) reported an unchanged disk halo size at 3 months after EVO ICL implantation. This discrepancy may be attributed to high myopia and preoperative spectacle correction. These findings indicate that PIOL correction potentially mitigates halo symptoms in patients compared with the correction achieved using glasses.

The static and dynamic contrast sensitivity tests also revealed no significant differences between the PR and ICL eyes for all SFs. Additionally, no significant differences were observed in wavefront aberrations between the two groups pre- and post-surgery. However, in both groups, trefoil for the 5 mm pupil diameter significantly increased post-surgery (both *P* < 0.05), possibly linked to the surgical incision (26). Similarly, Wan et al. (27) noted an increase in trefoil at 3 and 6 months post-ICL implantation. Previous studies have shown that the ICL induces fewer ocular HOAs than LRS (20, 28). Our study found that the HOAs introduced by the PR and ICL were comparable.

At 1 year after PR implantation, the mean MTF cutoff was 30.08 cpd, mean SR was 0.17, and mean OSI was 1.15. In the ICL group, the mean MTF cutoff was 34.99 cpd, mean SR was 0.20, and mean OSI was 0.95. These results of ICL are consistent with earlier findings. He et al. (29), Jiang et al. (30) reported that 3 months after EVO ICL implantation, the mean MTF cutoff was 31.23 and 38.81 cpd, mean SR was 0.17 and 0.25, and mean OSI was 0.99 and 0.87, respectively. Comparatively, the OSI in the PR group was higher than that in the ICL group, and the PR group exhibited lower values of MTF cutoff, SR, OV100%, OV20%, and OV9% than those in the ICL group. Significant differences in the MTF cutoff and SR were observed between the two groups. Overall, the ICL group demonstrated superior OQAS results compared with the PR group. This outcome may be attributed to several factors. First, differences in materials may account for the optical variations between the two lenses. Second, our observations during the slit-lamp examinations revealed greater lens pigmentation in the PR group than that in the ICL group, potentially leading to increased scattering. The higher hardness of the PR materials may have contributed to greater pigment dispersion.

Our study had certain limitations. First, the sample size was small. A larger sample is required to comprehensively assess the safety, efficacy, and visual quality of PR implantation. Second, the absence of pre-surgical measurements of OQAS scores made it challenging to pinpoint the specific cause of the differences between the two groups, necessitating further investigation into pre- and post-PR implantation changes. Thirdly, the EVO + model offers larger optical zones, potentially leading to improved quality of vision, particularly regarding night symptoms. The EVO model was utilized in our study due to the unavailability of the EVO + model in mainland China.

Phakic refractive implantation was safe and effective for myopia correction with good predictability and stability. Notably, the PR demonstrated a lower postoperative vault than the ICL. PR had better nighttime visual quality in the early postoperative period, perhaps due to the larger optical area; however, there was no significant difference between PR and ICL in the glare/halo at 1 year postoperatively. By further optimizing the power and size calculation formulae, PR implantation can correct myopia more accurately and achieve an ideal vault. Therefore, the PR is a novel option for PIOL applications.

## Data Availability

The raw data supporting the conclusions of this article will be made available by the authors, without undue reservation.

## References

[1] Martínez-Plaza E López-de la Rosa A López-Miguel A Holgueras A Maldonado MJ. EVO/EVO+ visian implantable Collamer lenses for the correction of myopia and myopia with astigmatism. *Expert Rev Med Devices.* (2023) 20:75–83. 10.1080/17434440.2023.2174429 36708714

[2] Montés-Micó R Ruiz-Mesa R Rodríguez-Prats J Tañá-Rivero P. Posterior-chamber phakic implantable Collamer lenses with a central port: A review. *Acta Ophthalmol.* (2021) 99:e288–301. 10.1111/aos.14599 32841517 PMC8246543

[3] Moshirfar M Webster C Ronquillo Y. Phakic intraocular lenses: An update and review for the treatment of myopia and myopic astigmatism in the United States. *Curr Opin Ophthalmol.* (2022) 33:453–63. 10.1097/ICU.0000000000000870 35916572

[4] Miao H Zhao F Niu L Zhao J Wang X Zhou X. One-step viscoelastic agent technique for ICL V4c implantation for myopia. *Int J Ophthalmol.* (2021) 14:1359–64. 10.18240/ijo.2021.09.10 34540611 PMC8403858

[5] Chen X Han T Zhao F Miao H Wang X Zhou X. Evaluation of disk halo size after implantation of a Collamer lens with a central hole (ICL V4c). *J Ophthalmol.* (2019) 2019:7174913. 10.1155/2019/7174913 31485347 PMC6710753

[6] Zhao W Han T Li M Sekundo W Aruma A Zhou X. Nighttime symptoms after monocular Smile: A contralateral eye study. *Ophthalmol Ther.* (2021) 10:1033–44. 10.1007/s40123-021-00396-5 34559401 PMC8589907

[7] Zhao W Zhao J Han T Li M Wang J Zhou X. Evaluation of disk halo size and identification of correlated factors in myopic adults. *Front Med.* (2022) 9:743543. 10.3389/fmed.2022.743543 35155490 PMC8831374

[8] Miao H Tian M Xu Y Chen Y Zhou X. Visual outcomes and optical quality after femtosecond laser small incision lenticule extraction: An 18-month prospective study. *J Refract Surg.* (2015) 31:726–31. 10.3928/1081597X-20151021-01 26544559

[9] Miao H He L Shen Y Li M Yu Y Zhou X. Optical quality and intraocular scattering after femtosecond laser small incision lenticule extraction. *J Refract Surg.* (2014) 30:296–302. 10.3928/1081597X-20140415-02 24893354

[10] Miao H Tian M He L Zhao J Mo X Zhou X. Objective optical quality and intraocular scattering in myopic adults. *Invest Ophthalmol Vis Sci.* (2014) 55:5582–7. 10.1167/iovs.14-14362 25103263

[11] Tan Q Lin J Tian J Liao X Lan C. Objective optical quality in eyes with customized selection of aspheric intraocular lens implantation. *BMC Ophthalmol.* (2019) 19:152. 10.1186/s12886-019-1162-6 31319806 PMC6639902

[12] Martínez-Roda J Vilaseca M Ondategui J Giner A Burgos F Cardona G Optical quality and intraocular scattering in a healthy young population. *Clin Exp Optomet.* (2011) 94:223–9. 10.1111/j.1444-0938.2010.00535.x 21083759

[13] Liao X Lin J Tian J Wen B Tan Q Lan C. Evaluation of optical quality: Ocular scattering and aberrations in eyes implanted with diffractive multifocal or monofocal intraocular lenses. *Curr Eye Res.* (2018) 43:696–701. 10.1080/02713683.2018.1449220 29630420

[14] Miao H Chen X Tian M Chen Y Wang X Zhou X. Refractive outcomes and optical quality after implantation of posterior chamber phakic implantable collamer lens with a central hole (ICL V4c). *BMC Ophthalmol.* (2018) 18:141. 10.1186/s12886-018-0805-3 29898694 PMC6001218

[15] Jonker S Berendschot T Saelens I Bauer N Nuijts R. Phakic intraocular lenses: An overview. *Indian J Ophthalmol.* (2020) 68:2779–96. 10.4103/ijo.IJO_2995_20 33229653 PMC7856940

[16] Packer M. Evaluation of the EVO/EVO+ sphere and Toric visian icl: Six month results from the United States food and drug administration clinical trial. *Clin Ophthalmol.* (2022) 16:1541–53. 10.2147/OPTH.S369467 35645557 PMC9132105

[17] Kamiya K Shimizu K Igarashi A Kobashi H Ishii R Sato N. Clinical evaluation of optical quality and intraocular scattering after posterior chamber phakic intraocular lens implantation. *Invest Ophthalmol Vis Sci.* (2012) 53:3161–6. 10.1167/iovs.12-9650 22661546

[18] Hyun J Lim D Eo D Hwang S Chung E Chung TY. A comparison of visual outcome and rotational stability of two types of toric implantable Collamer lenses (TICL): V4 versus V4c. *PLoS One.* (2017) 12:e0183335. 10.1371/journal.pone.0183335 28846701 PMC5573270

[19] Packer M. The implantable Collamer lens with a central port: Review of the literature. *Clin Ophthalmol.* (2018) 12:2427–38. 10.2147/OPTH.S188785 30568421 PMC6267497

[20] Wei R Li M Zhang H Aruma A Miao H Wang X Comparison of objective and subjective visual quality early after implantable Collamer lens V4c (ICL V4c) and small incision lenticule extraction (SMILE) for high myopia correction. *Acta Ophthalmol.* (2020) 98:e943–50. 10.1111/aos.14459 32419383

[21] Sachdev G Singh S Ramamurthy S Rajpal N Dandapani R. Comparative analysis of clinical outcomes between two types of posterior chamber phakic intraocular lenses for correction of myopia and myopic astigmatism. *Indian J Ophthalmol.* (2019) 67:1061–5. 10.4103/ijo.IJO_1501_18 31238411 PMC6611309

[22] Li B Chen X Cheng M Lei Y Jiang Y Xu Y Long-term vault changes in different levels and factors affecting vault change after implantation of implantable Collamer lens with a central hole. *Ophthalmol Ther.* (2023) 12:251–61. 10.1007/s40123-022-00606-8 36335511 PMC9834492

[23] Schmidinger G Lackner B Pieh S Skorpik C. Long-term changes in posterior chamber phakic intraocular Collamer lens vaulting in myopic patients. *Ophthalmology.* (2010) 117:1506–11. 10.1016/j.ophtha.2009.12.013 20363503

[24] Puell M Pérez-Carrasco M Barrio A Antona B Palomo-Alvarez C. Normal values for the size of a halo produced by a glare source. *J Refract Surg.* (2013) 29:618–22. 10.3928/1081597X-20130819-03 24016347

[25] Zhao W Zhao J Han T Wang J Zhang Z Zhou XA. comprehensive investigation of contrast sensitivity and disk halo in high myopia treated with SMILE and EVO implantable Collamer lens implantation. *Transl Vis Sci Technol.* (2022) 11:23. 10.1167/tvst.11.4.23 35452094 PMC9055559

[26] Kim S Yang H Yoon G Lee Y Kweon M Kim J Higher-order aberration changes after Implantable Collamer lens implantation for myopia. *Am J Ophthalmol.* (2011) 151:653–662.e1. 10.1016/j.ajo.2010.10.031 21310383

[27] Wan T Yin H Wu Z Yang Y. Comparative study of implantable Collamer lens implantation in treating four degrees of myopia: Six-month observation of visual results, higher-order aberrations, and amplitude of accommodation. *Curr Eye Res.* (2020) 45:839–46. 10.1080/02713683.2019.1701690 31801031

[28] Chen X Guo L Han T Wu L Wang X Zhou X. Contralateral eye comparison of the long-term visual quality and stability between implantable collamer lens and laser refractive surgery for myopia. *Acta Ophthalmol.* (2019) 97:e471–8. 10.1111/aos.13846 30187653 PMC6585688

[29] He T Zhu Y Zhou J. Optical quality after posterior chamber Phakic implantation of an intraocular Lens with a central hole (V4c implantable Collamer Lens) under different lighting conditions. *BMC Ophthalmol* (2020) 20:82. 10.1186/s12886-020-01340-0 32131800 PMC7055093

[30] Jiang Z Wang H Luo D Chen J. Optical and visual quality comparison of implantable Collamer lens and femtosecond laser assisted laser in situ keratomileusis for high myopia correction. *Int J Ophthalmol.* (2021) 14:737–43. 10.18240/ijo.2021.05.15 34012890 PMC8077018

